# Smart Sensing System for the Prognostic Monitoring of Bone Health

**DOI:** 10.3390/s16070976

**Published:** 2016-06-24

**Authors:** Nasrin Afsarimanesh, Asif I. Zia, Subhas Chandra Mukhopadhyay, Marlena Kruger, Pak-Lam Yu, Jurgen Kosel, Zoltan Kovacs

**Affiliations:** 1 School of Engineering and Advanced Technology, Massey University, Palmerston North 4442, New Zealand; afsarimanesh@yahoo.com (N.A.); a.i.zia@me.com (A.I.Z.); P.Yu@massey.ac.nz (P.-L.Y.); 2 Department of Engineering, Macquarie University, North Ryde NSW 2109, Australia; 3 Institute of Food Science and Technology, Massey University, Palmerston North 4442, New Zealand; M.C.Kruger@massey.ac.nz; 4 Sensing, Magnetism and Microsystems Group, King Abdullah University of Science and Technology, Thuwal 23955-6900, Saudi Arabia; jurgen.kosel@kaust.edu.sa; 5 Department of Physics and Control, Faculty of Food Science, Szent István University, Budapest H-1118, Hungary; zoltan.kovacs@chemometrics.hu; 6 Department of Physics, COMSATS Institute of Science and Technology, Islamabad 45550, Pakistan

**Keywords:** bone turnover markers, interdigital sensors, Enzyme-Linked Immunosorbent Assay (ELISA), Electrochemical Impedance Spectroscopy (EIS)

## Abstract

The objective of this paper is to report a novel non-invasive, real-time, and label-free smart assay technique for the prognostic detection of bone loss by electrochemical impedance spectroscopy (EIS). The proposed system incorporated an antibody-antigen-based sensor functionalization to induce selectivity for the C-terminal telopeptide type one collagen (CTx-I) molecules—a bone loss biomarker. Streptavidin agarose was immobilized on the sensing area of a silicon substrate-based planar sensor, patterned with gold interdigital electrodes, to capture the antibody-antigen complex. Calibration experiments were conducted with various known CTx-I concentrations in a buffer solution to obtain a reference curve that was used to quantify the concentration of an analyte in the unknown serum samples. Multivariate chemometric analyses were done to determine the performance viability of the developed system. The analyses suggested that a frequency of 710 Hz is the most discriminating regarding the system sensitivity. A detection limit of 0.147 ng/mL was achieved for the proposed sensor and the corresponding reference curve was linear in the range of 0.147 ng/mL to 2.669 ng/mL. Two sheep blood samples were tested by the developed technique and the results were validated using enzyme-linked immunosorbent assay (ELISA). The results from the proposed technique match those from the ELISA.

## 1. Introduction

Bone is an active tissue that undergoes continuous remodeling, the process of replacing old bone tissues by new tissues. The activity of osteoclasts (bone resorption) and osteoblasts (bone formation) are responsible for this process [1]. During childhood and early adulthood years, bone formation dominates, so bones become denser, heavier, and larger. This condition continues until the age of 30 years, when bones reach their maximum density and strength (peak bone mass). Under normal conditions, bone resorption and bone formation are coupled with each other to provide a balance in skeletal metabolism and turnover [1]. The health condition of bone stays quite stable during the ages of 30–45 years depending on lifestyle, but at later age, the bone resorption process (the activity of osteoclasts) begins to exceed the bone formation rate, consequently causing a loss in bone density and deterioration in microarchitecture that leads to fractures and osteoporosis. Osteoporosis is one of the world’s most common diseases that results in the bones becoming porous and fragile [2]. In women, bone loss is relatively faster in the first years after menopause [3,4]. Osteoporosis affects both genders, but women are more at risk of developing it. Statistically speaking, one in three women, and one in five men, above 50 years of age experience osteoporotic fractures in their life [5,6]. Therefore, this disease is a serious public health concern for postmenopausal women and aged people [4]. The female hormone, estrogen, is essential for having healthy bones. After menopause, the level of this hormone falls and this can lead to a quick decrease in bone density [3]. With the escalating trend in the geriatric population and growing healthcare costs, inexpensive prognostic and diagnostic monitoring of bone modeling and remodeling processes in older people is of utmost importance.

Type I Collagen is the principal structural protein of bones which forms approximately 90% of the organic bone matrix [1]. During the process of bone resorption, type I collagen is broken down and C-terminal telopeptide of type I collagen (CTx-I) is secreted into the bloodstream [7]. Hence, CTx-I is a biochemical marker that can be used for the measurement of bone resorption and can give information about the rate of bone turnover [8], where the reference range of CTx-I level in a normal person is between 0.040 ng/mL and 0.630 ng/mL [9]. Currently, biochemical bone markers, such as C-terminal telopeptide of type I collagen (CTx-I) and N-terminal telopeptide of type I collagen (NTx-I) are used as a clinical research tool, but these markers are not measured routinely in the same patient. Since the biochemical markers vary from day to day, or week to week, frequent measurement of the level of biochemical markers may help to monitor bone loss or the response to treatment for osteoporosis.

Most of the available techniques to detect and measure biochemical and bone turnover markers are based on the enzyme-linked immunosorbent assay (ELISA). ELISA is an analytical tool that is widely used in biomedical research for the detection and measurement of a specific antigen in a liquid sample. ELISA uses enzyme-linked antigens and antibodies to detect the target molecule. Very small quantities of antigens, such as hormones, proteins, peptides, or antibodies in a liquid sample can be detected using ELISA [10,11]. The antigen in the liquid phase is coated into the wells of a 96-well microtiter plate that binds to a primary antibody. A secondary, enzyme-linked antibody then detects the antigen by binding the antigen to the antibody. A chromogenic substrate is used to change the color in the presence of the antigen. Finally, the measurement is done using spectrophotometry [12]. Though this method is a standard immunoassay technique and has been commercialized, there are some drawbacks in using ELISA; it is a laboratory-based assay that is time-consuming, expensive, and requires several steps and technical expertise. It involves numerous steps and procedures for incubation, antibody binding, and measurements that requires not only services of highly-skilled professionals and an expensive laboratory setup, but also involves high costs for testing individual samples and, hence, cannot be used for frequent monitoring of the CTx-I levels to track changes in bone resorption in an individual.

Since changes in bone mineral density (BMD) is very slow, BMD studies are required to be of longer intervals between the measurements, at least two years, while changes in biochemical markers may be identified after only a few weeks [13]. It will be very important to have a sensing system which can frequently monitor the effect of bone loss in blood or urine, if possible. The bone loss usually results in higher amounts of CTx-I in the blood. Hence, continuous detection of CTx-I by a sensing system can provide useful information about bone health. Measurement of BMD, along with the detection and measurement of biochemical markers, can aid in the monitoring of osteoporosis and the response to treatment.

Planar interdigital sensors are made of a comb-like periodic pattern of parallel electrodes on a rigid substrate. One of the most important benefits of the planar interdigital sensors is the single-side access to the sample. This property helps to penetrate the sample with magnetic, electric, or acoustic fields from one side only. The sensor can be optimized to control the strength of the output signal by changing the sensing area, the number of interdigital electrodes, and the spacing between the electrodes to carry useful information as the signal emerges out of the biological sample [14]. The capability of being used for non-destructive testing is another advantage of these sensors, which makes them more useful for in vitro testing and process control applications [15]. High penetration depth planar interdigital sensors have been designed and fabricated with a greater number of sensing electrodes as compared to the excitation electrodes, in order to increase the penetration depth of the fringing electric field. When a time-dependent electrical signal is applied to the excitation electrode of the sensor, an electric field is generated which travels from the excitation electrode to the sensing electrode. The alternating electric field penetrates through the test-sample via the excitation electrode and is received by the sensing electrode that carries useful information about the properties of the material under test in close proximity of the sensor. Different geometries have been studied in the research literature [16,17,18]. Planar interdigital sensors have been used in numerous applications, such as detection of DNA [19], phthalates in water and juices [20], and *Staphylococcus aureus* [21]. Several sensing systems were developed based on the high penetration depth interdigital sensors for the detection of dangerous contaminated chemicals in seafood [22], environmental monitoring [18], and phthalate detection in aqueous solutions [20]. Thus, it would be useful to have an electrochemical biosensor at a relatively low cost, as a point-of-care tool to establish a realistic measurement profile for patients. In this research work, electrochemical impedance spectroscopy (EIS) was used for the detection and quantification of CTx-I using a functionalized interdigital sensor. The current EIS system exhibits a sensitivity of 26.063 per ng/mL. EIS is a label-free technique that is extremely sensitive to the interfacial binding events occurring at the binding sites of the biosensitive probe making an impedimetric electrochemical biosensor particularly advantageous for the rapid real-time measurements [23]. Among several methods available for impedance measurements, a frequency response analyzer (FRA) has become a de facto standard for EIS. FRA is a single sinusoidal wave input method in which a low amplitude AC sine wave of a given frequency is applied to the excitation electrode and the resulting AC current is measured. The system remains pseudo-linear at a low amplitude AC signal. The process could be repeated for the desired frequency range, and impedance is calculated for five to ten different measurements per decade change in frequency. The EIS data can be graphically presented as Bode and Nyquist (Cole-Cole) plots. These plots show the electrochemical interactions occurring at the interface of the electrode and electrolyte. Several applications of EIS have been reported, such as modeling the behavior of supercapacitors [24,25], the determination of the corrosive behavior of materials [26], the detection of Afbiotoxin B1 (AFB1) in olive oil [27], phthalates in water and juices [20], cancer cells [28], human immunoglobulin A [29], fat contents in meat [30], biotoxins in shell fish [16], bacterial endotoxin in food [31], and analysis of electrical properties of soy milk [32]. Immunosensors for impedimetric detection of bone biomarkers have been reported by other groups [23,33]. The functionalization of a silicon-substrate sensor has been previously achieved [33] with a self-assembled monolayer (SAM) of dithiodipropionic acid, streptavidin, and biotinylated antibody to determine the changes in impedimetric readings on the attachment of type I collagen. A three-electrode system was used in this process. However, the procedure was convoluted as they used a lot of reagents for the functionalization of the sensor. There was no information about any single optimal frequency that could give the highest sensitivity to the system. Moreover, real samples should be tested using the developed sensing system and results should be validated using a commercial device. Another group has reported an impedimetric detection technique for bone biomarkers [23] by functionalizing gold-coated carbon nanotube electrodes with Avidin and biotinylated antibody. However, testing with real samples are yet to be validated.

## 2. Materials and Methods

The main contribution of the current research is to design, develop, and experimentally validate a novel sensor for continuous monitoring of bone loss. The system is developed on a MEMS-based planar interdigital sensor with selective coating materials on the sensor surface. The coating material is used to detect the C-terminal telopeptidetype one collagen (CTx-I). The development of the complete system is described step-by-step in the following sections.

### 2.1. High Penetration Depth Planar Interdigital Sensor

**Figure 1:**
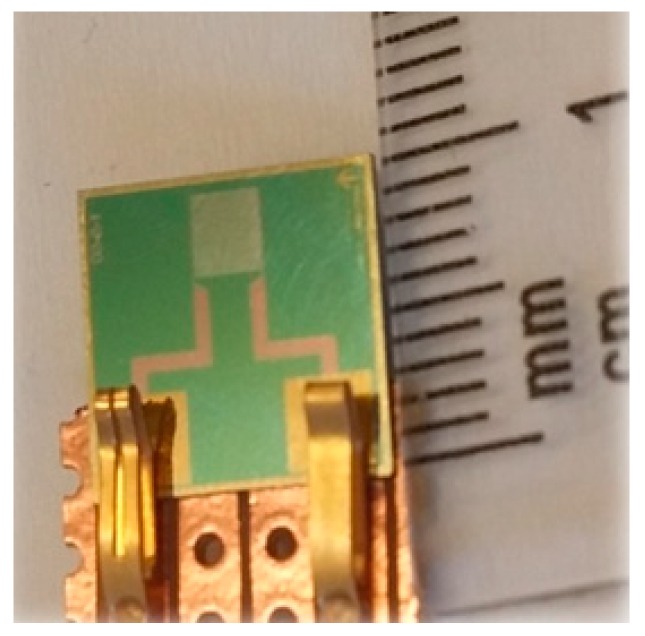
Sensor’s design and connection pads.

High penetration depth planar interdigital sensors, as shown in Figure 1, were used to perform the experiments. These sensors were fabricated in a clean room facility at King Abdullah University of Science and Technology (KAUST), Thuwal, Saudi Arabia, consequent to an academic collaboration between the School of Engineering and Advanced Technology, New Zealand, and KAUST.

The higher penetration depth was achieved by increasing the number of sensing (working) electrodes between the reference electrodes. A time-dependent sinusoidal electrical perturbation is applied to the excitation electrodes of the interdigital sensor. The switching electric field bulges through the test-sample via the excitation electrode and is received by the sensing electrode, which carries useful information about the properties of the material under test in close proximity of the sensor.

The fabrication of the sensors was carried out using photolithography and etching techniques on a single crystal silicon/silicon dioxide (Si/SiO_2_) wafer with a thickness of 525 µm and diameter of 4 inches. Thirty-six workable sensors were fabricated on one wafer; each sensor has a dimension of 10 mm × 10 mm and sensing area of 2.5 mm × 2.5 mm. Gold was used as the electrode material due to the flexibility in the methods available for its deposition as thin film electrodes and also due to its inertness, excellent physiochemical stability, bio-affinity, low electrical resistivity, and minimal oxidation risk.

A sensor with the configuration of 1-11-50 was used for the experiments, which suggests that there are eleven sensing electrodes present between two excitation electrodes having a distance of 50 µm between two consecutive electrodes.

### 2.2. Materials and Chemicals

The Serum CrossLaps^®^ ELISA kit is a product of IDS Company (Boldon, UK), was purchased locally from Abacus ALS, Auckland, New Zealand. The kit contained a streptavidin-coated microtiter plate, biotinylated antibody, peroxidase conjugated antibody, six known concentration antigen solutions, washing buffer, incubation buffer, and stopping solution. Known concentration antigen standards, biotinylated antibody, and peroxidase conjugated antibody from an ELISA kit were also used for performing the experiments using the developed sensing system. Streptavidin agarose was procured from Sigma-Aldrich, St. Louis, MO, USA.

### 2.3. Experiments Using the Serum CrossLaps^®^ ELISA Kit

**Figure 2:**
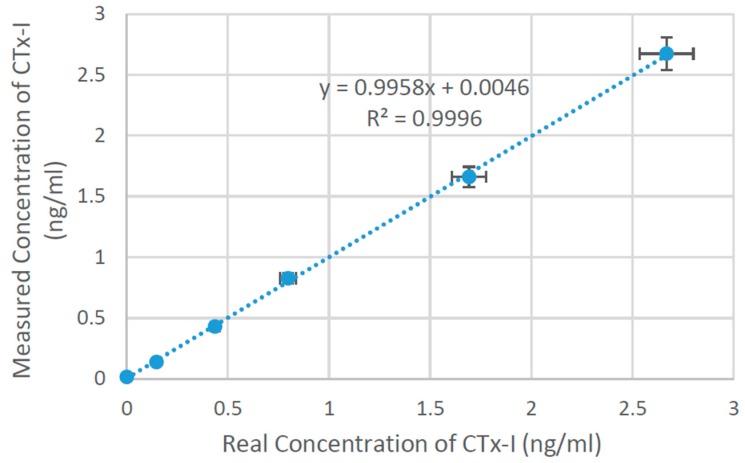
The standard curve plotted from the ELISA results.

The Serum CrossLaps^®^ ELISA kit was used to obtain the standard curve to validate the results obtained from the developed technique by testing the available known concentration antigen solutions. The Serum CrossLaps^®^ ELISA is an enzyme immunological standard test for quantification of CTx-I in plasma. There are different types of ELISA available [10], the Serum CrossLaps^®^ ELISA is based on the sandwich method. The steps were followed according to the standard ELISA procedure. Figure 2 shows the standard curve obtained from ELISA. All samples were tested in duplicate and the assay was performed at room temperature. Once the standard curve was obtained the experiments were performed to measure CTx-I concentration in two unknown samples that were obtained from sheep blood.

### 2.4. Coating of Sensor for Inducing Selectivity

**Figure 3:**
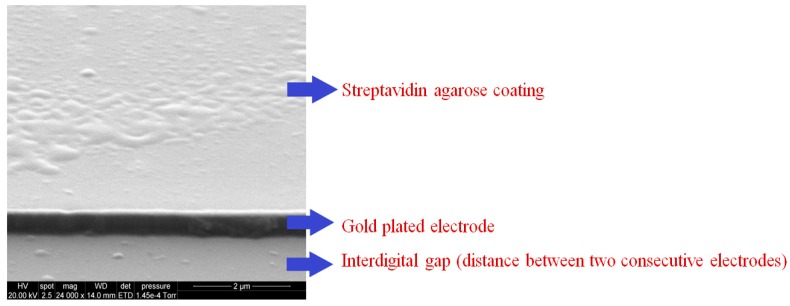
SEM image of streptavidin agarose-coated sensing surface.

The sensing area of the sensor was spin-coated with 4 µL of streptavidin agarose in order to functionalize the sensing surface [34]. The sensor was dried under nitrogen atmosphere and was later characterized again by impedance spectroscopy to determine the change in impedance profile, which was compared to that of the uncoated sensor in order to obtain a reference plot for the individual interdigital sensor. Figure 3 shows the SEM image of streptavidin agarose-coated sensor. Streptavidin agarose acts as a cross-linker between the gold electrodes/SiO_2_ substrate and biotinylated CTx-I antibodies that are responsible for capturing the analyte from serum or urine samples. At the next stage, the antibody-antigen solution was prepared by mixing the antigen, biotinylated antibody and a peroxidase-conjugated antibody which are available in the ELISA kit. The prepared solution was then incubated for an hour to allow antibodies to entrap CTx-I molecules from the test sample, before pipetting 8 µL of the solution on the streptavidin-coated sensing surface of the interdigital sensor.

Later, one-hour incubation at room temperature was allowed for streptavidin-coating to cross-link the antibody-antigen complex onto the gold interdigital electrodes. The sensor was washed five times with a washing buffer solution and dried under nitrogen at room temperature. Finally, the profiling of the sensor was carried out with the LCR (inductance (L), capacitance (C), resistance (R) meter and the impedance was evaluated using EIS technique.

**Figure 4:**
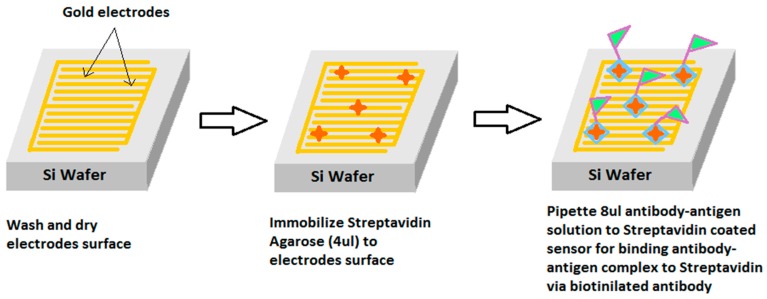
Graphical illustration of the steps required to prepare the sensing surface for CTx-I sensing.

Figure 4 shows the schematic of the steps required to prepare the biosensing surface for the selective binding of CTx-I molecules on the electrode surface.

### 2.5. Experimental Setup and Electrochemical Impedance Spectroscopy

**Figure 5:**
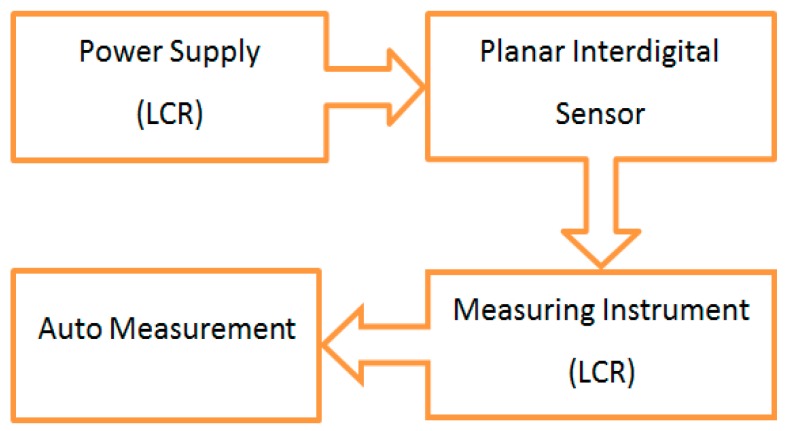
Block diagram of the measurement acquisition system.

The experimental setup consisted of a thermometer and a humidity meter, interdigital sensor and material under test, and a high-precision LCR meter (Hioki 3532-50, LCR Hi Tester, Nagano, Japan) that was connected to the computer through the RS232 port. The device provided the power, as well as monitored the changes in the impedance reading from the sensor. The sensor was connected to the reference and working probes (crocodile clip terminal probes) via gold pin connectors. The block diagram of the measurement acquisition system is shown in Figure 5.

The bare interdigital sensor was EIS profiled in air in order to characterize the sensor and to determine the sensitive frequency range for the specific sensor. There was no visible change at frequencies less than 100 Hz. The range of operating frequencies is basically dictated by the LCR meter used in the laboratory. A HIOKI 3532-50 LCR meter provides the operating frequency range from 42 Hz to 5 MHz.

## 3. Results and Discussions

### 3.1. CTx-I Measurement in Known Samples Using the Proposed Sensing System

**Figure 6:**
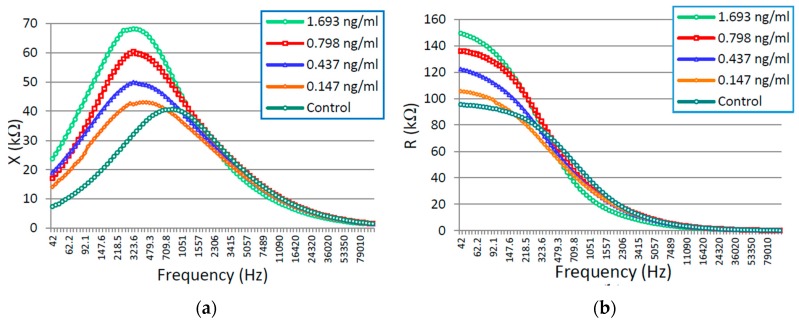
(**a**) Imaginary part (reactance) of the impedance vs. frequency; and (**b**) real part of the impedance vs. frequency.

Samples with four known concentrations (0.147 ng/mL, 0.437 ng/mL, 0.798 ng/mL, and 1.693 ng/mL) were tested in the developed sensing system. The standard solution with zero concentration of CTx-I was considered as the control. Experiments were conducted at room temperature (21 °C) at a humidity level of 31%. Tests on the samples were performed by the developed sensing system immediately after preparing the sample solutions. Figure 6a shows the reactance in the frequency domain for all four CTx-I concentrations. As illustrated in this figure, the capacitive reactance (X) shows clear changes, especially at lower frequencies between 100 Hz and 750 Hz, with a change in CTx-I concentration that is attributed to the dielectric properties of the sample. The real part of impedance vs. frequency for different concentrations of CTx-I is plotted in Figure 6b. The change in the resistive part of the impedance (R) is seen only at very low frequencies up to 150 Hz, which is mainly due to the ionic properties and faradic current through the sample material. The sensitivity obtained from the reactance part is also higher than the resistive part. Therefore, the reactance was used to evaluate the concentration of CTx-I in the sample solutions.

**Figure 7:**
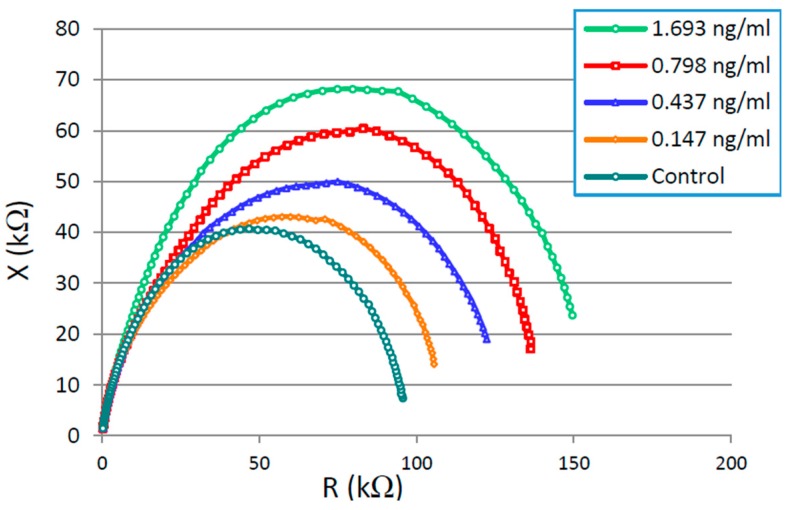
Nyquist plot for different CTx-I concentrations.

Figure 7 shows the Nyquist plot for the impedance spectrum obtained for all four concentrations of CTx-I in a frequency range of 42 Hz to 100 kHz. It was observed that the diameter of the semicircle increases by increasing the concentration of CTx-I depicting the increase in charge transfer resistance due to the presence of the higher amount of CTx-I attached to the sensing surface.

### 3.2. Data Analyses Using Non-Linear Least Square Curve Fitting

The equivalent circuit was deduced by applying a complex non-linear least square method (CNLS) that fits the experimentally-observed impedance spectrum on theoretically-evaluated values for an electrical circuit. It interprets the electrochemical kinetic processes executing inside a chemical cell into its electrical equivalent circuit based on Randle’s model [15,35].

**Figure 8:**
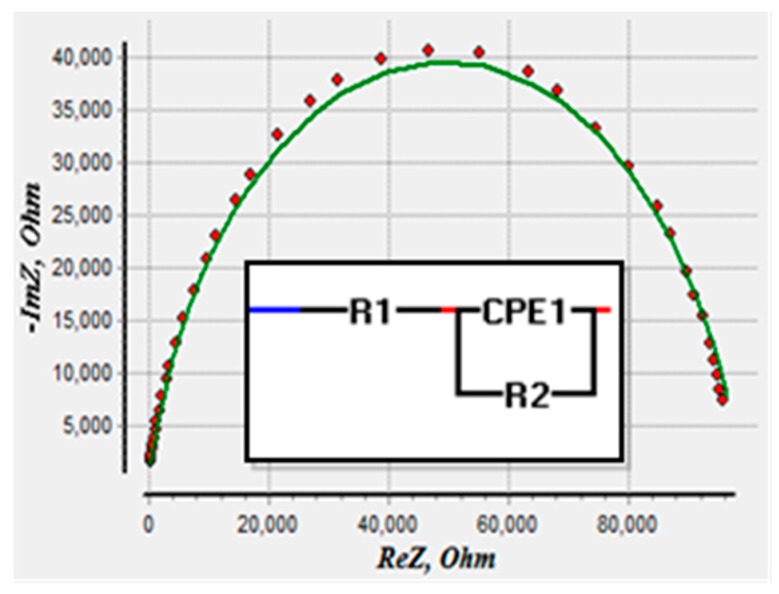
Proposed equivalent circuit by CNLS with a parallel combination of constant phase element (CPE1) and charge transfer resistance (R2) in series with the solution resistance (R1).

EIS Spectrum analyzer algorithm was used to estimate the equivalent circuit. The fitted Nyquist plot and the proposed equivalent circuit for the electrochemical processes are given in Figure 8, where the points on the graph represent the experimentally-observed data and the solid line shows the theoretically-fitted response for the equivalent circuit. The equivalent circuit proposed by the complex non-linear least square curve fitting is a parallel combination of a constant phase element (CPE1) and charge transfer resistance (R2) in series with the solution resistance (R1).

Table 1 displays the estimated component parameters and values of the equivalent circuit. P1 and n1 are parameters of constant phase element, representing the pre-exponential factor and exponent, respectively. The fitted value of n1 dictates the capacitive behavior of CPE1 as shown in Table 1 [36]. The evaluation error was <2.8% for the equivalent circuit parameters. ${r_{amplitude}}^{2}$ indicates the deviation of the experimentally-observed value from the optimal solution.

### 3.3. Multivariate Chemometric Analyses

The results of impedance spectroscopy measurements were subjected to multivariate data evaluation. Principal component analysis (PCA) was used to identify possible outliers and to discover the multidimensional patterns of the individual parameters namely, the real part of impedance R (Ohm), reactance X (Ohm), and phase shift (degree). Results of PCA showed that the highest variation related to the concentration change of CTx-I occurs in the lower frequency range in the case of all three measured parameters. Therefore, the frequency range between 42 and 5917 Hz was used for further chemometric evaluation. PCA models were calculated using the truncated frequency range for all the three individual parameters, respectively. The first two principal components (PC1 and PC2) described more than 98% of the whole variation in all three models. Furthermore, PC1 and PC2 presented good separation of the groups of the different concentration CTx-I samples.

**Figure 9:**
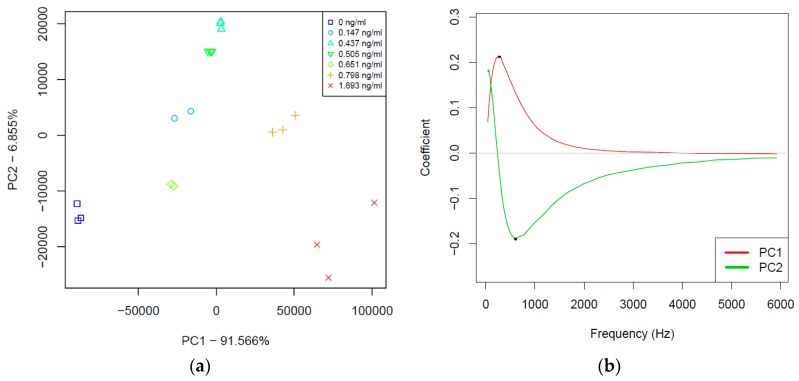
(**a**) Principal component analysis score plot (PC1-PC2); and (**b**) principal component analysis loadings plot (PC1-PC2).

PCA results of the reactance (X) parameter are shown in Figure 9a,b. A PCA score plot (PC1 and PC2) of reactance (X) were calculated based on the frequency range between 42 and 5917 Hz. Figure 9a demonstrated a tendency of separation of the different concentration CTx-I samples based on their increasing concentration. The loadings of PCA model in the Figure 9b implied the importance of the frequency range between 42 and about 2000 Hz in this separation.

Following the PCA calculations, regression models were built with partial least square regression using the three individual parameters (Rs, X, and phase angle), separately and using the frequency range between 42 and 5917 Hz to regress on the CTx-I concentration.

**Figure 10:**
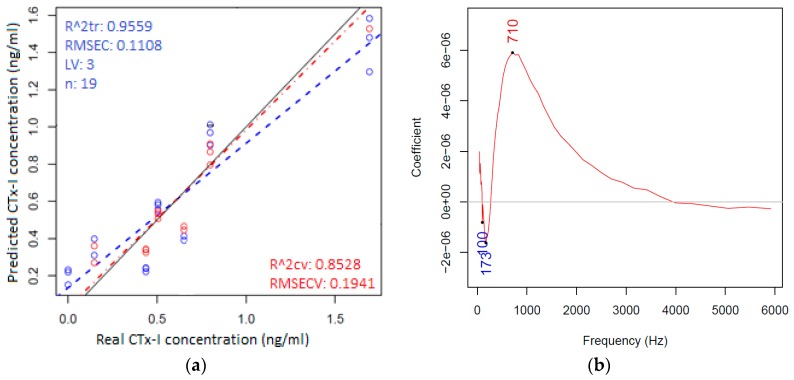
(**a**) Partial least square regression model to regress on the CTx-I concentration (red color is for calibration and the blue color is for cross-validation model); and (**b**) regression vector of the partial least square regression model showing 710 Hz as the most discriminating frequency.

The best regression model was found for reactance (X) data as shown in Figure 10a,b. The PLSR model of reactance (X) parameter provided relatively close correlation and a low prediction error. Results of cross-validation showed the concentration of CTx-I samples could be predicted based on reactance (X) with an error of 0.1941 ng/mL in the concentration range between 0 and 1.693 ng/mL using the frequency range between 42 and 5917 Hz. The regression vector of the PLSR model (Figure 10b) emphasizes the highest importance of 710 Hz for the regression of CTx-I concentration.

### 3.4. CTx-I Measurement in Unknown Samples Using the Proposed Sensing System

Based on the results from multivariate analyses the sensitivity of the sensor was calculated at a frequency of 710 Hz from the reactance (X) data using the following equation:

(1) $$Sensitivity\;\left(\%\right)\;=\;\frac{Z_{imag}\left(Control\right)-\;Z_{imag}\left(Sample\right)}{Z_{imag}\left(Control\right)}\;\times 100$$

**Figure 11:**
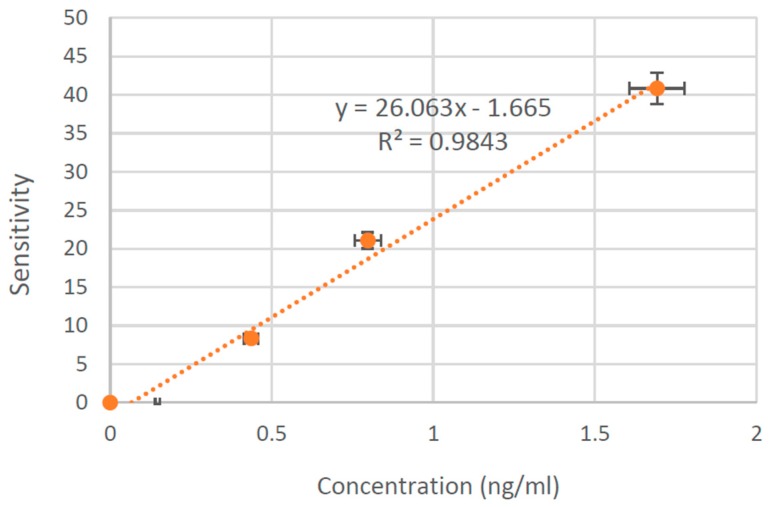
The reference calibration curve for the sensitivity of the sensor vs. concentration evaluated at 710 Hz.

A reference curve was obtained by plotting the sensitivity against the concentration, which is shown in Figure 11. The results of sensitivity are derived from the imaginary part of the impedance where the numerical value corresponds to 26.063 per ng/mL. This curve could be used to measure the concentration of CTx-I in any unknown sample with CTx-I concentration ranging between 0 ng/mL and 1.693 ng/mL. Once the reference curve was plotted, further experiments were performed in order to evaluate the CTx-I concentration for the two unknown samples of sheep blood that were evaluated using ELISA in order to validate the EIS system performance. The calculated concentration of CTx-I in the first unknown CTx-I concentration sheep blood sample was evaluated to be 0.62 ng/mL, whereas the calculated concentration of the peptide was 0.52 ng/mL in the second sample. The results obtained were validated using ELISA. The error for the first sample was 4.3% and, for the second one, was 4.6%.

The detection limit of the proposed sensor is 0.147 ng/mL and the corresponding reference curve is linear in the range of 0.147 ng/mL to 2.669 ng/mL. Three different concentrations of CTx-I less than 0.147 ng/mL were tested using the proposed sensing system; however, the sensor could not give accurate results for those samples.

## 4. Conclusions

A non-invasive, real-time, and label-free sensing technique towards early detection of bone loss by employing an EIS technique has been reported. The proposed system incorporated antibody-antigen-based functionalization by employing streptavidin agarose as a cross-linker for binding CTx-I peptides. A planar interdigital sensor has been used in conjunction with a FRA algorithm to measure the electrochemical impedance of the samples. Multivariate chemometric analyses have been performed to determine the single optimal frequency that could provide the highest sensitivity to the sensing system. Calibration experiments have been conducted with variously known CTx-I concentrations in a buffer solution to obtain a reference curve that was used to quantify the concentration of an analyte in the unknown serum sample. Two unknown samples, obtained from sheep blood, have been tested by the developed sensing system and the results were validated using ELISA.

This research work is a part of an on-going exploration, focusing on developing an inexpensive, robust, and portable sensing system. This has the potential to be used by the geriatric population in the domestic and point-of-care environment to monitor CTx-I levels in urine on a daily basis in order to avoid musculoskeletal disorders, like bone carcinoma and osteoporosis. To achieve robustness in the developed system ‘artificial antibodies’ are under extensive research by the group.

## Figures and Tables

**Table 1:** Equivalent circuit parameters.

Component Parameters	0.147 ng/mL	0.437 ng/mL	0.798 ng/mL	1.693 ng/mL
R1(Ω)	1.790 × 10^−14^	1.686 × 10^−14^	1.657 × 10^−14^	1.660 × 10^−12^
R2(Ω)	1.153 × 10^5^	1.347 × 10^5^	1.480 × 10^5^	1.596 × 10^5^
P1	1.535 × 10^−8^	1.588 × 10^−8^	1.036 × 10^−8^	6.464 × 10^−9^
n1	0.7944	0.7944	0.8345	0.8823
${r_{amplitude}}^{2}$	0.0133	0.0100	0.0161	0.0246
